# Screening and Probiotic Property Analysis of High Exopolysaccharide-Producing Lactic Acid Bacteria from Sayram Yogurt

**DOI:** 10.3390/microorganisms14010140

**Published:** 2026-01-08

**Authors:** Xudong Zhao, Kaiyue Wang, Zhaojun Ban, Jia Li, Xingqian Ye, Wei Liu, Xiaoyu Wang, Heng Xu, Heng Zhang, Hui Zhang, Zisheng Yang, Longying Pei

**Affiliations:** 1 Xinjiang Auricularia Engineering Technology Research Center, Experimental Teaching Demonstration Center of Food Safety and Nutrition, Xinjiang Institute of Technology, Aksu 843100, China; moreonce0@163.com (X.Z.); 18599112185@163.com (K.W.);; 2 College of Food Science and Engineering, Tarim University, Alar 843300, China; 3 School of Biological and Chemical Engineering, Zhejiang University of Science and Technology, Zhejiang Provincial Key Laboratory of Chemical and Biological Processing Technology of Farm Products, Zhejiang Provincial Collaborative Innovation Center of Agricultural Biological Resources Biochemical Manufacturing, Hangzhou 310023, China; banzhaojun@zust.edu.cn; 4 National-Local Joint Engineering Laboratory of Intelligent Food Technology and Equipment, Zhejiang Key Laboratory for Agro-Food Processing, Zhejiang Engineering Laboratory of Food Technology and Equipment, Fuli Institute of Food Science, College of Biosystems Engineering and Food Science, Zhejiang University, Hangzhou 310058, China

**Keywords:** Sayram yogurt, exopolysaccharide, lactic acid bacteria, isolation, screening, probiotics

## Abstract

Exopolysaccharides (EPSs) produced by lactic acid bacteria (LAB) are bioactive polymers with significant potential for human health. This study aimed to isolate and systematically evaluate the in vitro probiotic properties of high exopolysaccharide-producing LAB strains from traditional Sayram yogurt. From fifteen strains, six strains with high exopolysaccharide production were identified using 16Sr DNA sequencing. We assessed their probiotic potential by testing acid resistance, bile salt tolerance, tolerance to artificial gastrointestinal fluid, self-aggregation, hydrophobicity, safety, antibacterial activity, and antioxidant capacity. Results showed these six strains exhibited a strong tolerance to acid, bile salts, and artificial gastrointestinal fluids, and had high self-aggregation abilities and surface hydrophobicity. The isolated strains exhibited varying degrees of sensitivity to the tested antibiotics, with no hemolysis, suggesting good safety. In addition, their cell-free supernatants significantly inhibited the growth of *Staphylococcus aureus* and showed stronger antioxidant activity than cell lysates. In conclusion, the six LAB strains screened in this study possess excellent in vitro probiotic properties and have potential value for further development, providing a preliminary strain reserve and theoretical reference for subsequent research and related product development.

## 1. Introduction

Sayram yogurt is a traditional hand-fermented dairy product in Sayram Town, Baicheng County, Xinjiang. The product typically exhibits a pH value of 4.70, with acidity levels reaching up to 113.1 °T. When stored under refrigeration at 4 °C Sayram yogurt generally has a shelf life of 7 days. It exhibits a relatively high viscosity and assumes a gelatinous state [1]. It is an intangible part of the cultural heritage of the Uyghur ethnic group in Xinjiang, reflecting the local dietary culture, and is often referred to as the “secret to longevity” [2]. It is a product fermented by natural flora, including LAB and yeast [3,4]. Studies have shown that LAB have potential health benefits, including effects on immune regulation [5], intestinal flora balance [6], antioxidants [7], and antibacterial aspects [8]. The metabolic products of LAB include EPSs, organic acids, bacteriocins, and aromatic compounds [9], which play significant roles in promoting gut health and enhancing food preservation. Among them, EPS have attracted attention because of their roles in prebiotic properties and improving food quality [10].

EPSs produced by LAB are natural high-molecular-weight polymers secreted extracellularly during their growth [11]. They are either released like mucus or attached to the bacterial cell wall to form a capsule [12]. For LAB, EPSs can enhance stress resistance by forming a stable biofilm and help them colonize [13]. EPS exhibits various biological activities, including antibacterial activity [14], regulating intestinal health [15], providing antioxidant activity [16], providing anti-cancer activity [17], reducing cholesterol, and regulating immunity [18]. In the food industry, EPSs serve as natural thickeners, emulsifiers, and stabilizers, effectively modifying the rheological properties and sensory quality of food [19]. In biomedicine, the use of probiotics to prevent and manage biofilms on medical devices has demonstrated promise. The inhibition rate of EPSs on these biofilms can reach 88%, offering a novel environmentally friendly approach for addressing medical device-related infections. This strategy holds substantial research significance and practical potential, particularly given the escalating challenge of drug-resistant bacteria [20].

Although the research value of EPSs produced by LAB has been recognized, systematic screening within the scope of traditional fermented foods is still insufficient. This study aims to isolate LAB that exhibit high production of extracellular polysaccharides from traditional Sayram yogurt. Probiotic characteristics, safety, and in vitro antioxidant capacity were comprehensively evaluated to provide strain resources and offer a theoretical basis for their functional development and industrial utilization.

## 2. Materials and Methods

### 2.1. Materials and Reagents

The Sayram yogurt samples for the experiment were obtained from a local market in Sayram Town, Baicheng County, Aksu Prefecture, Xinjiang. Six independent samples were gathered, all prepared by local herdsmen using traditional fermentation methods. After collection, the samples were immediately placed in sterile bags, stored in vehicle-mounted low-temperature refrigerators, and swiftly transported to laboratory refrigerators set at 4 °C and −80 °C for storage.

We obtained the following: MRS (Beijing Land Bridge Technology Co., Ltd., Beijing, China); agar powder (Beijing Lanjeke Technology Co., Ltd., Beijing, China); Gram Staining Kit, dialysis bag (MWCO: 8000–14,000 Da), pepsin, and trypsin, which were obtained from Beijing Solarbio Science & Technology Co., Ltd., Beijing, China; Bacterial Genomic DNA Extraction Kit (Beijing TIANGEN Biotech Co., Ltd., Beijing, China); bovine bile salts (Shandong Keyuan Biochemical Co., Ltd., Heze, China); vancomycin and othermycin (Changde Beckman Biotechnology Co., Ltd., Changde, China); Columbia blood agar plate (Qingdao High-tech Industrial Park Hope Bio-technology Co., Ltd., Qingdao, China); Antioxidant Activity Assay Kit (Shanghai Yuanye Bio-Technology Co., Ltd., Shanghai, China). *Escherichia coli* (ATCC 8099), *Staphylococcus aureus* (ATCC 6538), and *Bacillus cereus* (ATCC 2) were purchased from Beiing Biobank Biotechnology Co., Ltd. Beijing, China. Ascorbic acid, Trichloroacetic acid, and LB medium were purchased from Thermo Fisher Scientific (China) Co., Ltd. Shanghai, China.

### 2.2. Isolation and Preliminary Identification of LAB

Dissolve 10 mL of Sayram yogurt in 90 mL of sterile 0.9% saline solution, then thoroughly mix to create a 10^−1^ bacterial suspension. Transfer 1 mL of this bacterial suspension to a tube containing 9 mL of sterile saline and repeat the serial dilution process to perform 10-fold gradient dilutions. Choose three suitable concentrations (10^−4^, 10^−5^, 10^−6^) and spread 100 μL of the bacteria solution on MRS agar medium. Anaerobic culture at 37 °C for 24 to 48 h. Examine colony color, morphology, and smoothness [21]. Select single colonies with varying morphologies and purify by streaking three times. Colonies that are purple in the Gram stain and negative in the contact enzyme test are preliminarily identified as LAB. Store these in a refrigerator at −80 °C with 50% glycerol for future use.

### 2.3. Strain 16S rDNA Identification

Genomic DNA from the strain was extracted following the instructions provided in the DNA rapid extraction kit. Universal primers tailored for bacterial studies were employed to amplify the 16S rDNA gene [22]. Specifically, the primers 27F (5′-GTTTGATCMTGGCTCAG-3′) and 1492R (5′-TACGGYTACCTTGTTACGACTT-3′) were used. The PCR mixture (50 µL) consisted of 2 µL of template DNA, 2 µL of each primer, 25 µL of Taq PCR Master Mix (Beijing TransGen Biotech Co., Ltd., Beijing, China.), and sterile water to reach the final volume. The PCR reaction program was set as follows: pre-denaturation at 98 °C for 2 min; subsequently, 30 cycles of amplification were carried out (unwinding at 98 °C for 10 s; annealing at 55 °C for 30 s; and extension at 72 °C for 1.5 min), and finally extension at 72 °C for 10 min, maintained at 15 °C. Post-amplification, products were analyzed on a 1% agarose gel in 1 × TBX buffer at 150 V. Bands were visualized under 302 nm UV light. Successfully amplified products were sent to a testing company (Xi’an Shenggong Bioengineering Co., Ltd., Xi’an, China) for sequencing. The sequencing results were compared to known sequences in the GenBank database using BLAST (https://blast.ncbi.nlm.nih.gov/Blast.cgi, accessed on 10 September 2025) [23] from the National Center for Biotechnology Information (NCBI) in the United States. Employing Mega 11 software, a phylogenetic tree was developed from these comparative findings.

### 2.4. Screening of Exopolysaccharide-Producing Strains

The EPS extraction method follows the approach by Tian et al. [24] The isolated strain was cultured for 24 h and inoculation volume was 2% (*v*/*v*) (cell concentration of 10^9^ CFU/mL) in MRS liquid medium with a pH of 5.5, incubated at 34 °C for 19 h under anaerobic conditions, and then centrifuged at 4 °C, 8000 rpm for 10 min to remove bacteria. Trichloroacetic acid (TCA) was added to the supernatant until the final concentration was 100 g/L; it was mixed thoroughly and allowed to stand at room temperature for 30 min, then centrifuged at 8000 rpm at 4 °C for 10 min to remove the protein precipitate. The precipitate was dissolved in distilled water, and impurities were removed by dialysis using a bag with a molecular weight cut-off of 8000–14,000 Da for 72 h, with water changes every 8 h. After dialysis, the volume was adjusted to the original level using distilled water to yield a crude polysaccharide solution, which was reserved for future use. The phenol-sulfuric acid method and glucose standard curve, y = 0.7061x − 0.0231 (R^2^ = 0.9977), where the abscissa was glucose content and the ordinate was the absorbance at OD 490 nm, were used to determine LAB strains with high EPS production.

### 2.5. Growth Characteristics of Strains Under Acidic Stress

Following the method established by Guesh et al. [25], the pH of MRS Broth was adjusted to 1.5, 2.5, 3.5, 4.5, and 5.5 using 0.1 mol/L hydrochloric acid. After sterilizing and cooling the broth, the isolated strains were cultured for 24 h and inoculated into MRS Liquid medium at an inoculation volume of 2% (*v*/*v*) (cell concentration of 10^9^ CFU/mL). Incubate the cultures at 37 °C for 24 h to assess acid resistance. Measure the OD600 nm absorbance values in triplicate, using normal MRS broth as a control.

### 2.6. Determination of Bile Salt Tolerance

We slightly modified Hosseny et al.’s [26] method to evaluate the bile salt tolerance of strains. The strain cultured for 24 h was inoculated into MRS broth containing bovine bile salt at a 2% (*v*/*v*) vaccination rate (with a cell concentration of 10^9^ CFU/mL). The mass concentrations of cow bile salt were 0.1%, 0.2%, 0.3%, 0.4%, and 0.5% (*w*/*v*), respectively. We incubated the culture at 37 °C for 24 h. We then measured the absorbance at an optical density (OD) of 600 nm, conducting each measurement in triplicate.

### 2.7. Tolerance to Simulated Artificial Gastrointestinal Fluids

The simulated gastrointestinal fluid was prepared following the method of Wang et al. [27] with modifications. To prepare the artificial gastric juice, dissolve 1 g of pepsin (3000 U/g) in 90 mL of 0.9% normal saline. Adjust the pH to 2.5, dilute to 100 mL, filter through a 0.22 μm membrane for sterilization, and store at 4 °C. For artificial intestinal fluid, dissolve 0.1 g of trypsin (2500 U/L) and 0.15 g of porcine bile salt in 90 mL of 0.9% normal saline. Adjust the pH to 8.0, dilute to 100 mL, and filter through a 0.22 μm membrane for sterilization. Store at 4 °C.

The strains were incubated at 37 °C for 24 h, followed by centrifugation at 4000 rpm for 10 min at 4 °C. The pellet was washed twice with sterile physiological saline. We adjusted the concentration of the test bacterial solution to 10^9^ CFU/mL. After combining 1 mL of the bacterial suspension with 9 mL of simulated gastric fluid, the mixture was agitated at 90 rpm while maintaining a temperature of 37 °C for a 3 h incubation period. Viable bacteria counts were measured at 0 and 3 h intervals via plate counting, with survival rates determined via Equation (1).

(1) $$Survival/\%=\frac{Survival\;of\;Bacteria\;in\;Artificial\;Gastric\;Juice\;After\;3\;h\;(CFU/mL)}{Survival\;of\;Bacteriain\;Artificial\;Gastric\;Juice\;After\;0\;h\;(CFU/mL)}\times 100\%\;\;\;$$

After simulated digestion, take 1 mL of the solution and culture it in 9 mL of artificial intestinal fluid at 37 °C with agitation at 90 rpm for 3 h. Employ plate counts to assess live bacterial populations at 0 and 3 h. Calculate the survival rate using Formula (2).

(2) $$Survival/\%=\frac{Viable\;bacterial\;count\;post\;3\;h\;intestinal\;fluid\;simulation\;(CFU/mL)}{Viable\;bacterial\;count\;post\;0\;h\;intestinal\;fluid\;simulation\;(CFU/mL)}\times 100\%$$

### 2.8. Self-Aggregation Capability

The self-aggregation ability was assessed using a modified version of the method by Ash et al. [28]. Initially, bacterial liquid cultured for 24 h (10^9^ CFU/mL) was centrifuged at 6000 rpm for 10 min to collect the bacterial cells. The precipitate was resuspended in an equal volume of sterile PBS (pH 7.2) and washed three times with sterile PBS. We adjusted the absorbance of the bacterial liquid to 0.8 ± 0.05. The solution was left to stand at room temperature, and the uppermost suspension was collected at 1, 3, and 6 h to measure the OD600 nm value. The self-aggregation rate was calculated using sterile PBS as the control, in accordance with Formula (3).

(3) $$Self-aggregation\;rate\;(\%)=(1-\frac{At}{A0})\times 100\%$$

At represents varying time points’ absorbance values, while A0 denotes the absorbance at 0 h.

### 2.9. Determination of Bacterial Surface Hydrophobicity

Following the method of Adesulu-Dahunsi et al. [29], the strain was cultured in MRS broth at 37 °C for 18 h. After adjusting the concentration of the bacterial liquid to 10^9^ CFU/mL, it was then centrifuged at 10,000 rpm at 4 °C for 10 min. The bacterial sediment should be collected and washed twice with sterile PBS. The bacterial suspension was resuspended and adjusted to OD600 nm = 1.0. We took 1.2 mL of the bacterial suspension and added 0.3 mL of n-hexane along with 1.2 mL of xylene, then mixed it thoroughly. We vortexed the mixture for 30 s and incubated it at room temperature for 30 min. While preserving the integrity of the oil layer, we measured the OD600 nm value of the lower aqueous phase.

(4) $$Cell\;surface\;hydrophobicity\;(\%)=(1-\frac{dt}{ds})\times 100\%$$

ds represents the OD600 nm value of the bacterial suspension before the xylene treatment, and dt represents the OD600 nm value of the aqueous phase after the xylene treatment.

### 2.10. Antibiotic Susceptibility Test

Antibiotic susceptibility tests were conducted using the drug-sensitive paper agar diffusion method [30]. A 100 μL bacterial solution, cultured overnight, was evenly spread on the surface of MRS solid medium. Once the solution was absorbed, the agar disk was placed on the Petri dish with sterile forceps. The plates were incubated at 37 °C for 24 h, after which the diameter of the bacterial inhibition zone was measured. In this study, we slightly modified the method proposed by Anisimova et al. [31] to perform sensitivity tests on isolated strains using eight antibiotics. In this regimen, the following antibiotics were prescribed: Tetracycline at 30 micrograms per tablet, kanamycin at 30 micrograms per tablet, ampicillin at 10 micrograms per tablet, gentamicin ranging from 10 ± 2.5 micrograms per tablet, erythromycin at 15 micrograms per tablet, chloramphenicol at 30 micrograms per tablet, clindamycin at 20 micrograms per tablet, and vancomycin at 30 micrograms per tablet.

### 2.11. Growth Curve Determination

Test the growth of high-yield EPS strains. Inoculate 2% bacterial solution into MRS broth and cultivate at 37 °C for 24 h. Measure the OD600 nm value every 2 h during cultivation. Use MRS broth as a blank control. Draw a growth curve [32] with the cultivation time as the abscissa and the absorbance (OD600 nm) as the ordinate.

### 2.12. Hemolytic Properties

The strains were assessed for hemolytic activity to evaluate bacterial safety [33]. The bacterial liquid that was cultured for 24 h was streaked on Colombian blood agar plates, and the hemolytic activity of the strain was evaluated after incubation at 37 °C for 24 h. The area around the colony showing a green zone indicates α-hemolysis, while the area showing a transparent zone indicates β-hemolysis. The colony with no obvious change is non-hemolytic (γ-hemolysis).

### 2.13. Determination of Bacteriostatic Activity

The determination of antibacterial activity was carried out by the agar diffusion method, referring to the method of Roy et al. [34], with slight modifications. Indicator bacteria (*Staphylococcus aureus*, *Escherichia coli*, and *Bacillus cereus*) were inoculated in LB broth and cultured at 37 °C, shaking at 160 rpm for 24 h. LAB were inoculated at 2% (*v*/*v*) in 10 mL sterile MRS broth, incubated at 37 °C for 12 h at 200 rpm, and then centrifuged at 8000 rpm for 10 min. The indicator bacteria were spread on LB agar plates at 1% volume. Once dried, we prepared 8 mm diameter holes with a sterile perforator and remove the agar from the holes. We added 100 μL of the centrifuged supernatant of LAB to each well, and we used an equal volume of sterile LB broth as the negative control. All operations were carried out under sterile conditions. After diffusing at 4 °C for 4 h, the samples were incubated at 37 °C for 24 h, and we observed the diameter of the inhibition zone.

### 2.14. Antioxidant Capacity of Strains In Vitro

#### 2.14.1. DPPH Free Radical Scavenging Ability

The Kao et al. [35] method, adapted, was applied to evaluate the strains’ DPPH antioxidant potential. To perform the test, 2.0 mL of the overnight culture bacterial suspension (10^9^ CFU/mL) was mixed with 2.0 mL of 0.2 mmol/L DPPH ethanol solution (prepared in anhydrous ethanol). We allowed the reaction to proceed at room temperature in the dark for 30 min. Then, we centrifuged the mixture at 8000 rpm for 10 min. We measured the absorbance of the supernatant at 517 nm. We calculated the DPPH radical scavenging activity using Formula (5).

(5) $$DPPH\;radical\;clearance\;(\%)=\frac{\mathrm{Ai}\;-\;A}{\mathrm{Ai}}\times 100\%$$

The absorbance of sample A is a mixture of 2 mL of DPPH-anhydrous ethanol solution and 2 mL of the sample. Ai measures the absorbance of a solution where the sample is replaced with an equal volume of distilled water.

Ascorbic acid was chosen as the positive control. A curve was plotted with the mass concentration of ascorbic acid on the *x*-axis and the radical scavenging rate of DPPH on the *y*-axis, resulting in the equation y = 0.0453x + 0.4737, R^2^ = 0.9997.

#### 2.14.2. ABTS^+^ Free Radical Scavenging Ability

To prepare the ABTS^+^ working solution, combine 2.5 mL of a 7 mmol/L ABTS solution with 2.5 mL of a 2.75 mmol/L potassium persulfate solution. Allow the mixture to sit at room temperature in the dark for 12 h. Adjust the absorbance of the ABTS^+^ solution at 734 nm to 0.700 ± 0.02 using anhydrous ethanol. Mix 500 μL of the bacterial suspension (at 10^9^ CFU/mL) with 1000 μL of ABTS^+^ reagent solution to form the sample group; combine 500 μL of PBS with 1000 μL of ABTS^+^ reagent solution for the control group. Allow both solutions to stand in darkness at ambient temperature for 10 min. Measure the absorbance [36] at 734 nm and calculate the clearance using Equation (6).

(6) $${\mathrm{ABTS}}^{+}\;Free\;radical\;clearance\;(\%)=\frac{A0\;-\;A1}{A0}\times 100\%$$

A1 indicates the sample’s absorbance; A0 signifies the control’s absorbance; Both of them were measured at 734 nm.

Ascorbic acid was chosen as the positive control. A curve was plotted with the mass concentration of ascorbic acid on the *x*-axis and the ABTS^+^ radical scavenging rate on the *y*-axis, resulting in the equation y = 0.0781x + 0.0302, R^2^ = 0.9992.

### 2.15. Statistical Analysis

Statistical analysis was conducted using SPSS (V23.0, IBM Corporation, Armonk, NY, USA) software, while Origin 2018 (OriginLab Corporation, Northampton, MA, USA) and Microsoft Excel (V2021, Microsoft Corporation, Redmond, DC, USA) were used to facilitate auxiliary plotting and calculations. The significance of the experimental data was assessed based on three independent repetitions, applying ANOVA with Duncan’s multiple comparison test, with a significance level established at α = 0.05.

## 3. Results and Discussion

### 3.1. Isolation and Identification of LAB

**Figure 1:**
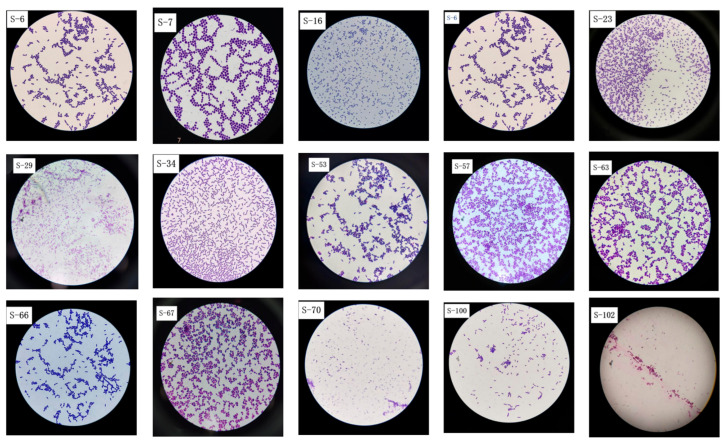
After Gram staining, 15 Sayram yogurt isolates underwent microscopic examination of colony morphology at 100× magnification.

Thirty-two suspected LAB colonies were initially isolated from the traditionally fermented Sayram yogurt samples. On the plate, the colonies appeared milky white or milky yellow and round. Most colonies were small, convex, moist, and viscous with smooth surfaces, while a few were larger. After Gram staining, 15 strains were identified as catalase-negative and Gram-positive. Under a light microscope, as shown in Figure 1, these strains appeared blue-purple, rod-shaped, spherical, and were either arranged side by side or scattered.

**Figure 2:**
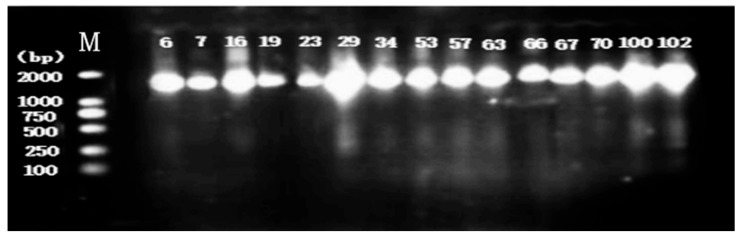
Agarose gel electrophoresis imaging was performed on the genomic DNA of 15 strains. Among them, M represents the marker, the number on the left represents the base pair number (bp) corresponding to the standard band, and the lane numbers correspond to the numbers of the 15 isolated strains.

DNA isolation and 16S rDNA gene PCR amplification were conducted on 15 presumptive LAB strains. The results of agarose gel electrophoresis (Figure 2) showed that all strains exhibited clear, specific bands at approximately 1000 bp, indicating that the amplification reaction was successful.

**Figure 3:**
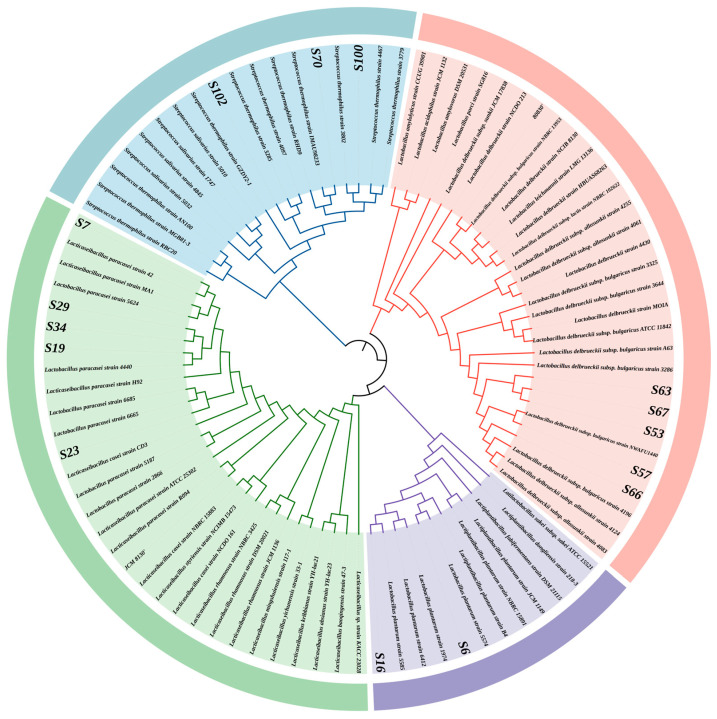
The phylogenetic analysis of the 16S rDNA gene sequences of the 15 isolated strains was carried out by MEGA 11 software using the maximum likelihood method. The end of each branch was colored according to the genus classification of the strains to visually display their taxonomic attribution.

The submitted 16S rDNA sequences underwent homology alignment in the NCBI database, where gene accession numbers were obtained. Subsequently, MEGA 11 software was employed to construct a phylogenetic tree. From the phylogenetic analysis depicted in Figure 3, the isolated strains were classified as follows: Strain S-6 was identified as *Lactiplantibacillus plantarum*; strains S-7, S-16, S-19, S-23, S-29, and S-34 were determined to be *Lacticaseibacillus paracasei*; strains S-53, S-57, S-63, S-66, and S-67 were identified as *Lactobacillus delbrueckii* subsp. *bulgaricus*; while strains S-70, S-100, and S-102 were found to be *Streptococcus salivarius* subsp. *thermophilus*. In total, 15 LAB strains were characterized.

### 3.2. Screening of High EPS-Producing Strains

**Figure 4:**
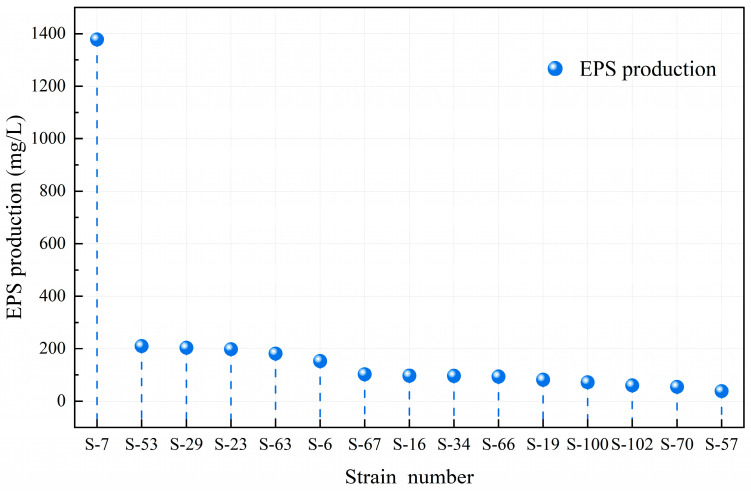
The ability of different LAB strains to produce EPSs. Different numbers correspond to the extracellular polysaccharide yield of each strain.

EPS is a beneficial compound produced by LAB during fermentation [37]. Different strains exhibit significant differences in EPS production capacity due to their specific structure [38]. Figure 4 shows that *Lacticaseibacillus paracasei* S-7 excels in exopolysaccharide synthesis, producing up to 1378.13 mg/L, far surpassing other strains. This indicates its superior viscosity-producing traits under identical culture conditions. *Lactobacillus delbrueckii* subsp. *bulgaricus* S-53 ranks second with 210.22 mg/L, followed by *Lacticaseibacillus paracasei* S-29 and S-23, yielding 203.61 mg/L and 198.41 mg/L, respectively. *Lactobacillus delbrueckii* subsp. *bulgaricus* S-63 produces 181.42 mg/L. The yield of *Lactiplantibacillus plantarum* S-6 was 153.09 mg/L. Other strains exhibit comparatively lower exopolysaccharide production. This study utilized the screening criteria established by Wa et al. [39]. in related research, setting an initial threshold of 150.00 mg/L for EPS yield to identify high-yield strains. Based on this criterion, six high-yield LAB (S-6, S-7, S-23, S-29, S-53, and S-63) were selected from the isolated strains, and their probiotic properties were subsequently assessed.

### 3.3. Growth Curve Assay

**Figure 5:**
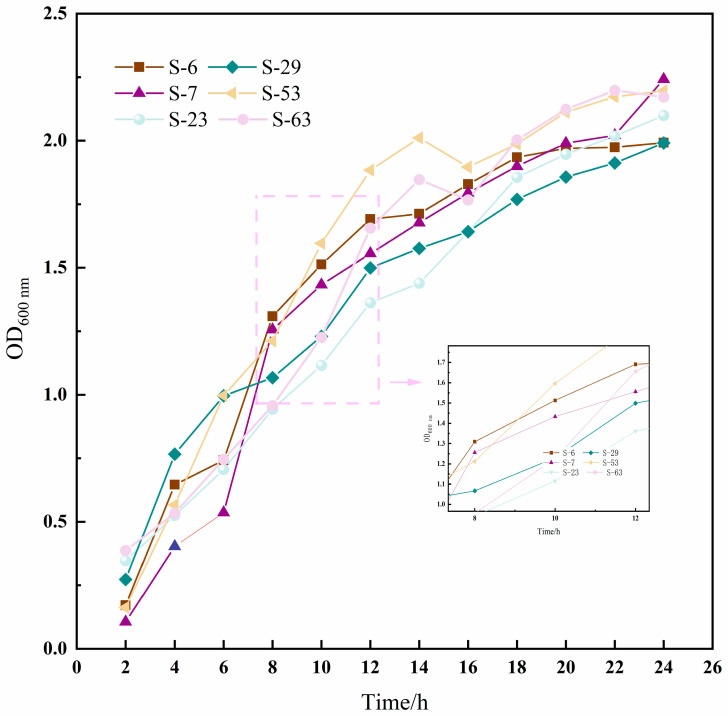
The growth kinetics curve of six strains of LAB in 24 h. The data were expressed as the absorbance (OD600) measured every 2 h, and the solid lines of different colors in the figure corresponded to the growth curves of each strain.

The growth curve can directly reflect the growth rate of the strain. In this study, six strains of LAB (S-6, S-7, S-23, S-29, S-53, and S-63) with high EPS yields were selected to monitor their growth dynamics. The results are shown in Figure 5; all strains displayed similar growth patterns. During the initial 2 h, the bacteria underwent a lag phase characterized by slow growth, as indicated by an OD600 nm value of approximately 0.2, suggesting adaptation to the new culture environment. After 4 h, the growth rate increased significantly, entering the logarithmic phase, where metabolic activity peaked. By 16 h, the growth rate began to decelerate, transitioning into a stationary phase. At this point, most strains stabilized with OD600 nm values exceeding 1.7.

### 3.4. Comprehensive Tolerance Analysis of Strains to Simulated Gastrointestinal Environment

**Figure 6:**
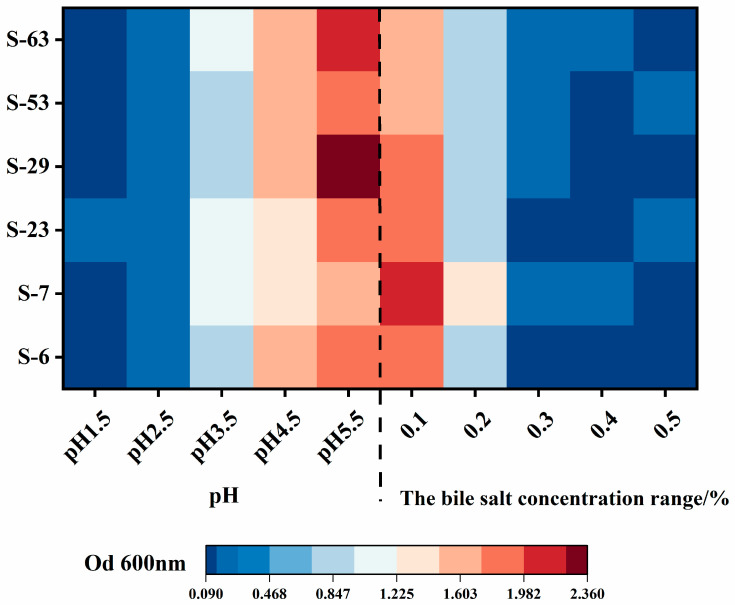
Acid and bile salt tolerance heat map of strains. The growth of six strains of LAB (S-6, S-7, S-23, S-29, S-53, S-63) under different pH (1.5–5.5) and different bile salt mass fraction (0.1%–0.5%) stress (expressed as OD600 nm value).

Human gastric juice typically has a pH ranging from 1.5 to 4.5 [40], and the ability of LAB strains to survive at pH levels between 2 and 4 is crucial [41] for their probiotic potential. The acid resistance of the isolated strains in this study is depicted in Figure 6. When the initial pH of the medium decreases, the OD600 nm values of each LAB strain decrease, and the acidic environment has a greater impact on their growth. When the initial pH is 5.5, all strains grow normally. When the pH drops to 4.5, the strain activity is weak but still relatively strong, and the OD600 nm exceeds 1.0. Under conditions of pH 3.5, the optical density at OD600 nm for each strain exhibits a decline, indicating that their growth is further inhibited. Among them, strain S-29 demonstrated the most pronounced decrease in OD, suggesting it possesses the weakest acid resistance. When the pH is reduced to extremely acidic levels of 2.5 or lower, the activity of the tested strains significantly diminishes and their growth is strongly inhibited. Monique et al.’s [42] research revealed that the 15 evaluated LAB isolates displayed tolerance to acidic conditions ranging from 2.0 to 3.0 pH. This performance was comparable to the acid tolerance observed in the strains screened in the present study under identical conditions. These findings suggest the evaluated strains possess the ability to survive in a low pH stomach environment.

As the main component of bile, bile salts can inhibit the growth and reproduction of bacteria by disrupting the structure and function of the LAB cell membrane and increasing its permeability [43]. Consequently, evaluating the tolerance of LAB to bile salts is essential for assessing its probiotic properties. Isolates that exhibit resistance to high concentrations of bile salts are more likely to survive and proliferate in the normal concentrations (0.03% to 0.3%) of bile found in the human gastrointestinal tract [44]. As illustrated in Figure 6, an increase in bile salt concentration (0.1–0.5%) resulted in concentration-dependent inhibition of growth activity across all LAB strains; specifically, higher bile salt concentrations correlated with reduced growth activity. At a bile salt concentration of 0.1%, the strains maintained high activity, with S-7 demonstrating the strongest performance. However, when the concentration of bile salts increased to 0.3%, the OD600 nm values for all strains significantly decreased, although they still exhibited some growth activity. At a high bile salt concentration of 0.5%, the strains displayed only minimal growth. The trend observed from OD600 nm measurements indicates that the inhibitory effect of bile salts on the growth of LAB is dose-dependent. Although the change in OD value is an indirect indicator, it is widely used in the preliminary screening of tolerance and is correlated with the trend of viable bacteria count [45]. Given that the bile salt content in the human intestinal tract is usually less than 0.3%, all the tested strains showed an increase in OD values at this concentration, preliminarily suggesting that they have the potential to survive in the intestinal environment. However, their colonization ability still needs to be further verified through direct methods such as CFU counting.

**Figure 7:**
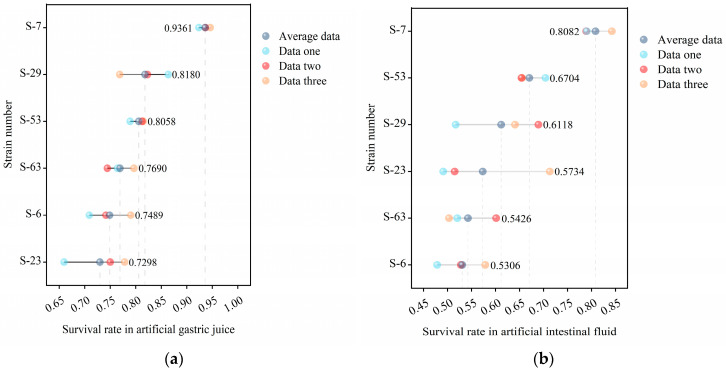
The gastrointestinal tolerance of 6 strains of LAB: (**a**) shows the survival rate in artificial gastric juice, and (**b**) shows the survival rate in artificial intestinal juice. The color points in the figure show 3 groups of repeated test data, and the gray points indicate the arithmetic mean of the survival rate of the corresponding strains.

The digestive environment of the gastrointestinal tract is a strong biological barrier. Gastric acid, digestive enzymes, and bile acids can degrade the microbial protein structure and exert pressure on it, thereby inhibiting or inactivating it and affecting foreign microorganisms [46]. The survival rate in the artificial gastrointestinal tract serves as a crucial indicator of the potential efficacy of probiotics [47]. Figure 7 illustrates the survival rates of LAB within the simulated gastrointestinal tract. All the tested strains performed well in the simulated gastrointestinal fluid, with a survival rate exceeding 50% after exposure. Notably, strain S-7 showed the highest resistance. Survival rates in simulated gastric and intestinal juices reached 93.61% and 80.82%, respectively. The survival rates of strains S-6, S-23, S-29, S-53, and S-63 in gastric juice were 74.89%, 72.98%, 81.80%, 80.58%, and 76.90%, respectively, and in intestinal juice, were 53.06%, 57.34%, 61.18%, 67.04%, and 54.26%, respectively.

Previous studies have demonstrated that EPS-producing LAB exhibit greater tolerance to bile salt environments compared to planktonic LAB [48], and the gastrointestinal tolerance of the strain is related to its ability to produce EPS [49]. The findings of this research align with the preceding assertions, which can be attributed to the fact that the EPSs produced by the strain are wrapped around the strain to form a protective layer, thereby enhancing its environmental tolerance.

### 3.5. Determination of Self-Aggregation Ability and Surface Hydrophobicity

**Figure 8:**
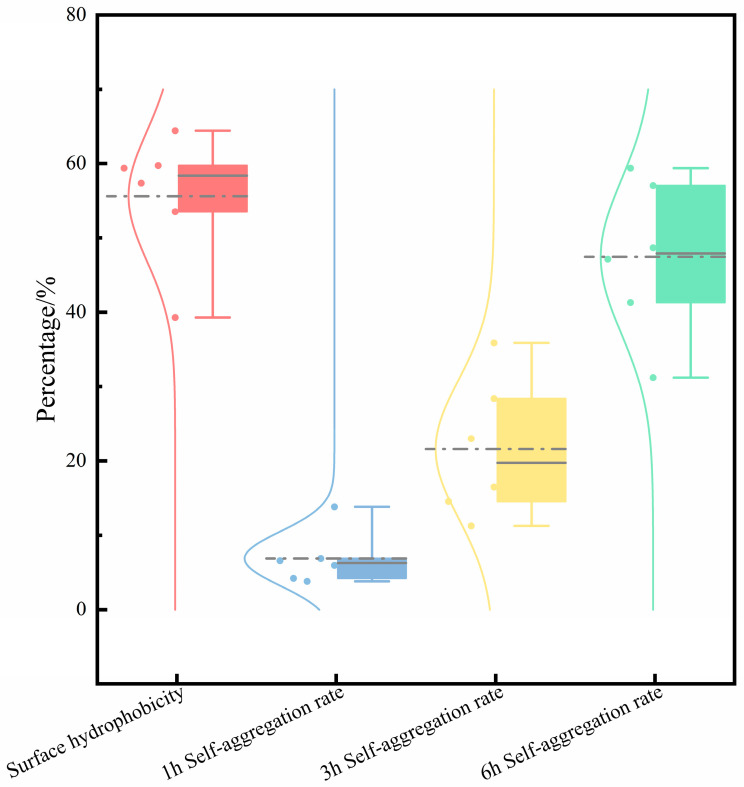
The surface hydrophobicity of six Lactobacillus strains along with their self-aggregation capabilities at 1, 3, and 6 h. Each box plot displays the interquartile range of the data, with the horizontal line inside representing the median value. The circular markers (•) and dashed lines indicate the mean values for each respective group.

Self-aggregation and hydrophobicity are key indicators of LAB’s non-specific adhesion to the intestinal tract [50]. Studies indicate that bacteria with high self-aggregation and surface hydrophobicity enhance colonization on the intestinal mucosa and promote adhesion to intestinal epithelial cells [51]. Probiotics maintain the balance of the flora by adhering to intestinal cells, occupying the colonization sites that pathogenic bacteria may use, thereby inhibiting infection [52]. In this study, we assessed the intestinal adhesion and colonization potential of the strains by measuring their self-aggregation ability and surface hydrophobicity. Figure 8 illustrates that all LAB strains exhibit a relatively strong aggregation ability, and the auto-aggregation rate shows an upward trend with time. After 6 h of culture, the aggregation rate ranges from 31.21% to 57.04%. The interaction between strongly hydrophobic bacteria and host cells is relatively strong, and they adhere more firmly to the intestinal and other mucosal surfaces [53]. Except for S-6, the hydrophobicity of each strain is between 39.30% and 64.04%, with S-6 exceeding 50%. All strains exhibit the auto-aggregation phenomenon, high adhesion, and the potential to adhere to intestinal epithelial cells, thereby demonstrating probiotic effects.

### 3.6. Assessment of Potential Safety Risks of LAB

**Figure 9:**
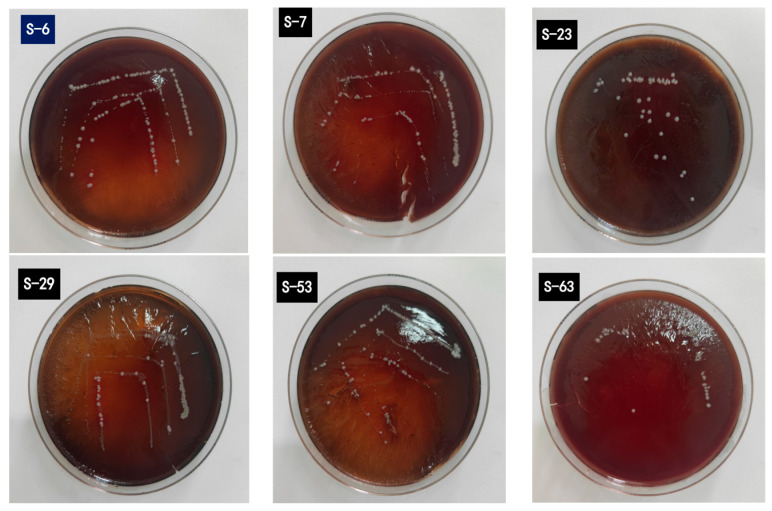
Illustrates the growth of six LAB strains on blood agar plates. No transparent (β-hemolysis) or grass-green (α-hemolysis) hemolytic rings were detected surrounding any of the colonies.

The World Health Organization and EFSA guidelines stipulate that probiotic strains must be devoid of hemolytic activity to ensure non-toxicity [54]. Hemolysis on blood agar plates is categorized into three types: α-hemolysis, indicated by green or tan rings around colonies; β-hemolysis, shown by pale yellow or clear circles, often linked to higher pathogenicity; and γ-hemolysis, which shows no change in the culture medium around colonies. In the hemolytic test of LAB, all tested strains had no occurrences of α- or β-hemolysis, thus confirming no hemolytic phenomena (Figure 9). The research results are the same as the previous report, and many probiotic LAB strains have no hemolytic activity in vitro [55].

Assessing the antibiotic resistance of probiotics is essential for identifying safe strains and preventing the transfer of resistance genes to intestinal pathogenic bacteria. This study assessed the susceptibility of LAB strains to eight common clinical antibiotics using the disk diffusion method to evaluate their safety. Table 1 shows that strains S-53 and S-23 were completely sensitive to all antibiotics. Strain S-29 exhibited high sensitivity to chloramphenicol, ampicillin, erythromycin, and gentamicin, as well as moderate sensitivity to clindamycin, kanamycin, tetracycline, and vancomycin. Conversely, isolates S-6, S-7, and S-63 exhibited high resistance to kanamycin, vancomycin, tetracycline, and gentamicin, with intermediate resistance to other tested antibiotics. The resistance of strains S-6, S-7, and S-63 to kanamycin and vancomycin aligns with the previous literature, which reports that many LAB species are highly resistant to these antibiotics [56]. Probiotics resist antibiotics through two main mechanisms: (1) natural or inherent resistance, which is non-transferable, and (2) acquired resistance, often due to bacterial mutations, which may transfer plasmids encoding antibiotic resistance genes to pathogens or other symbiotic bacteria [57]. Studies indicate most probiotics naturally resist kanamycin and vancomycin, with these resistances likely encoded in their chromosomes, making them non-inducible and non-transferable between bacteria. The observed tetracycline resistance in some strains may result from natural resistance mechanisms. Gentamicin, an aminoglycoside antibiotic, inhibits protein synthesis; however, Gram-positive bacteria, due to their thick cell walls, resist it because the antibiotic cannot penetrate to disrupt cell membrane synthesis [58]. The LAB isolates in this study showed a good state of safety through in vitro experiments, which lays a key foundation for their future applications as starters or probiotic cultures.

Inhibiting pathogens is key to selecting probiotics, because probiotic LAB produce antibacterial substances such as organic acids, H_2_O_2_, and bacteriocins during growth [60]. These metabolites compete with probiotics for adhesion sites and nutrients and can destroy and inhibit the colonization of pathogens [61]. In this study, the agar diffusion method was used to evaluate the antibacterial activity of six LAB strains against three kinds of bacteria: *Staphylococcus aureus*, *Bacillus cereous*, and *Escherichia coli*. Table 2 shows that all LAB strains can inhibit *Staphylococcus aureus* and *Bacillus cereous*. The inhibitory effect on *Staphylococcus aureus* is relatively obvious, and the diameter of the inhibitory zone is ≥15 mm. Previous studies confirmed that the *Lactiplantibacillus plantarum* strain can inhibit *Staphylococcus aureus* [62], and the results of this study support this conclusion. For *Bacillus cereous*, the antibacterial activities of strains S-6, S-7, and S-63 are relatively high, while the other strains are relatively weak. In contrast, no discernible inhibition zone was observed against *Escherichia coli* in any of the strains, aligning with previous reports that suggest LAB strains typically exhibit greater inhibitory activity against Gram-positive bacteria [63].

### 3.7. Antioxidant Ability of Strain In Vitro

**Figure 10:**
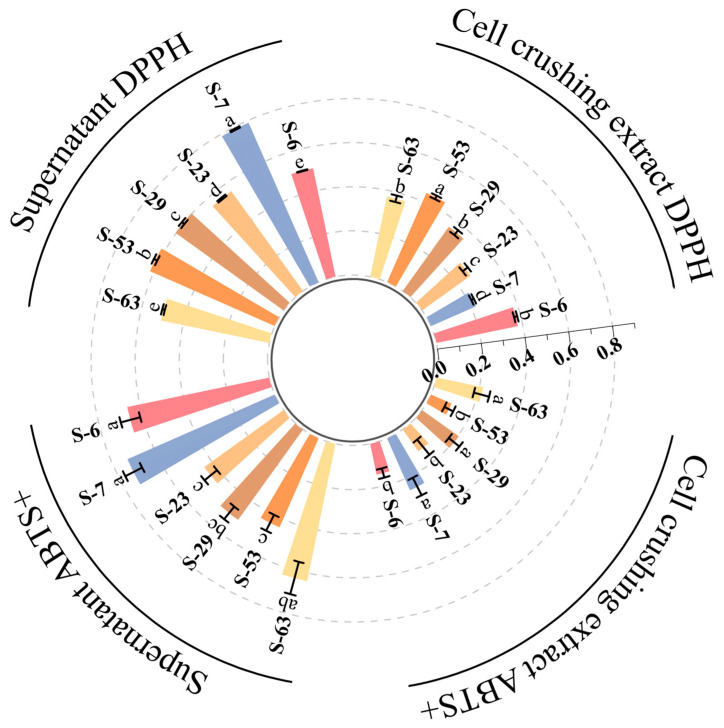
Evaluation of antioxidant activity of six strains of LAB. The radial bar graph compared the DPPH radical scavenging rate and ABTS^+^ radical scavenging rate of the cell lysate of the strain with the cell-free supernatant. Columns of different colors represent different strains. The data is expressed as the mean ± standard deviation of three repeated experiments, different letters represent significant differences between the two groups.

Antioxidant capacity is of great significance for alleviating oxidative stress. The antioxidant capacity of the isolated strains was evaluated by DPPH assay and ABTS assay, with ascorbic acid as the positive control. The results are shown in Figure 10. The cell-free supernatants and cell lysates of strains S-6, S-7, S-23, S-29, S-53, and S-63 all exhibited certain antioxidant activities. However, differences in antioxidant capacity were observed among the various strains (*p* < 0.05). Notably, the scavenging rates of DPPH free radicals in the cell-free supernatants of all six strains exceeded 50%. In particular, the cell-free supernatant of strain S-7 exhibited the strongest antioxidant activity, with a DPPH free radical scavenging rate reaching 78.91%. Its antioxidant activity is equivalent to that of 6.95 μg/mL of ascorbic acid. The cell disruption extract of S-53 is in the leading position in this regard, achieving a 44.14% yield. ABTS^+^ has a characteristic absorption peak at 734 nm and has a blue-green color. When adding antioxidants, they react with ABTS^+^ cation radicals, resulting in a color change and a decrease in absorbance. This change directly indicates the ABTS^+^ radical scavenging ability of the sample [65]. In comparison to other tested strains, the cell-free supernatant and cell disruption extract of strain S-7 exhibited the highest scavenging ability for ABTS free radicals, with scavenging rates of 72.81% and 25.34%, respectively. Through the evaluation of the free radical scavenging effect of ABTS^+^, it can be known that the antioxidant activity of the cell-free supernatant of strain S-7 is equivalent to that of 8.93 μg/mL ascorbic acid. This finding is comparable to the antioxidant capacity of the fermentation supernatant derived from LAB strains identified by Yang et al. [66] in the gastrointestinal tract of black goats in Hainan. In conclusion, six strains of bacteria have relatively strong extracellular antioxidant activity. The antioxidant activity of the cell-free supernatant is better than that of the cell disruption extract. This indicates that the main antioxidant substances are present in the extracellular secretions. Based on the EPS production data for each strain in this study (153.09 mg/L–1378.13 mg/L), it can be preliminarily inferred that the antioxidant capacity may be associated with the secreted EPSs. Research conducted by Adesulu-Dahunsi et al. [67] further demonstrated that in vitro antioxidant activity increased with higher EPS concentrations; no notable discrepancies were found across the EPS samples. Additionally, the activity exhibited a dose-dependent increase as EPS concentration rose. This finding aligns with the existing literature indicating that extracellular polysaccharides from LAB possess strong antioxidant properties [68]. Although this study shows that EPSs produced by LAB have considerable in vitro antioxidant activity, thorough and detailed food safety assessments are essential for the effective implementation of these strains in the food industry. In addition to in vitro antioxidant data, it is equally important to further verify the actual efficacy and mechanism of EPS in complex food processing systems and organisms through cell models and animal experiments.

## 4. Conclusions

Six strains of LAB with high EPS production were isolated from traditional Sayram yogurt. The EPS production levels for strains S-6, S-7, S-23, S-29, S-53, and S-63 were 153.09 mg/L, 1378.13 mg/L, 198.41 mg/L, 203.61 mg/L, 210.22 mg/L, and 181.42 mg/L, respectively. These strains exhibit potential probiotic properties: they adapted well to acidic and bile salt environments, tolerated artificial gastrointestinal fluid (>53%), and exhibited a certain degree of self-aggregation (31–57%) and hydrophobicity (39–64%). They were sensitive to certain antibiotics, lacked hemolytic properties, and inhibited *Staphylococcus aureus* and *Bacillus cereus* to varying degrees. The cell-free supernatant showed a DPPH scavenging rate over 50% and an ABTS^+^ free radical scavenging capacity of at least 43%. In conclusion, the LAB strains isolated and screened in this study demonstrate certain in vitro probiotic properties and exhibit potential for applications, thereby offering a theoretical reference for the development of related probiotic resources. However, the conclusion of this study is only based on in vitro experiments. The in vivo probiotic function of the strains, the specific structures of the produced EPSs, and the structure–activity relationship still need to be clarified. In the future, it is necessary to further verify the strains’ physiological activity in vivo through animal experiments. Additionally, by integrating EPS structure analysis with whole genome sequencing, the probiotic functional mechanism and biological safety of this strain should be revealed in detail, providing a more robust theoretical foundation and technical support for the industrial application of this strain and the development of functional products.

## Figures and Tables

**Table 1:** Antibiotic sensitivity of strains.

Strain Number	Antibiotics							
	Ampicillin (AMP)	Chloramphenicol (C)	Kanamycin (K)	Clindamycin (CC)	Tetracycline (TE)	Erythromycin (E)	Vancomycin (VA)	Gentamycin (GM)
S-6	I	I	R	I	R	I	R	R
S-7	I	I	R	I	R	I	R	R
S-23	S	S	S	S	S	S	S	S
S-29	S	S	S	S	S	S	S	S
S-53	S	S	S	S	S	S	S	S
S-63	I	I	R	I	R	I	R	R

Note: The criteria for interpreting drug sensitivity results adhere to the CLSI guidelines [59] and are classified as follows: R—drug resistance; I—intermediary; S—sensitive.

**Table 2:** Antibacterial activity of strains.

Pathogenic Bacteria	S-6	S-7	S-23	S-29	S-53	S-63
*Staphylococcus aureus*	+++	+++	+++	+++	+++	+++
*Bacillus cereous*	+++	+++	+	+	+	+++
*Escherichia coli*	-	-	-	-	-	-

Note: Antibacterial activity classification follows the criteria established by Tan Xiqian et al. [64], which categorizes using the following: (-) means no obvious inhibition zone, (+) means the inhibition zone is less than 5 mm, and (+++) means the inhibition zone is greater than 12 mm.

## Data Availability

The original contributions presented in this study are included in the article. Further inquiries can be directed to the corresponding author.
